# Mapping Environmental Suitability for Malaria Transmission, Greece

**DOI:** 10.3201/eid1905.120811

**Published:** 2013-05

**Authors:** Bertrand Sudre, Massimiliano Rossi, Wim Van Bortel, Kostas Danis, Agoritsa Baka, Nikos Vakalis, Jan C. Semenza

**Affiliations:** European Centre for Disease Prevention and Control, Stockholm, Sweden (B. Sudre, M. Rossi, W. Van Bortel, J.C. Semenza);; Hellenic Centre for Disease Control and Prevention. Athens, Greece (K. Danis, A. Baka);; National School for Public Health, Athens (N. Vakalis)

**Keywords:** Malaria, infectious diseases, transmission, Greece, parasites, vector-borne infections, mapping

## Abstract

During 2009–2012, Greece experienced a resurgence of domestic malaria transmission. To help guide malaria response efforts, we used spatial modeling to characterize environmental signatures of areas suitable for transmission. Nonlinear discriminant analysis indicated that sea-level altitude and land-surface temperature parameters are predictive in this regard.

Malaria was eliminated in Greece in 1974 (*1*,*2*); however, cases continue to be imported from countries to which malaria is endemic (*3*) and locally acquired cases have occurred sporadically (*4*,*5*). During 2009–2012, health authorities in Greece recorded 267 malaria cases. Although most cases were imported, at least 69 (26%) occurred in patients who did not have travel histories to malaria-endemic regions. A cluster of 6 locally acquired *Plamosdium vivax* malaria cases occurred during August–October 2009 in the southern Peloponnese (Evrotas Municipality, Lakonia district); in addition, 1 autochthonous case was reported from Marathon Municipality, East Attiki district (*2*). In 2010, locally transmitted cases were recorded in the same Lakonia district, 1 in East Attiki and 2 in children in central Greece (Viotia district). In 2011, a total of 42 autochthonous cases of *P. vivax* malaria were reported, representing 44% of the 96 notified cases in 2011. Most (36) of those cases were notified in the Evrotas municipality. In 2012, locally acquired cases appeared to have decreased, with 16 cases representing 21% of the overall number of cases. The ongoing transmission of *P. vivax* by local anopheline mosquitoes raises concern about reemergence of malaria transmission in Greece in areas that are hospitable to the vector and have permissive environmental and climatic conditions (*6*).

## The Study

To guide malaria control efforts, we delineated areas suitable for malaria transmission in Greece using the place of exposure for 69 locally acquired malaria cases. A health officer administered a standardized questionnaire to each malaria case-patient in Greece to determine the origin of infection. Our analysis was restricted to cases classified as locally acquired in persons without travel histories to optimize the specificity of the model because our goal was to describe the environmental suitability of autochthonous malaria transmission in Greece. Thus, our analysis excluded cases that might have been acquired abroad.

We aimed to describe the environmental profile of areas with active transmission cycles during 2009–2012 and then to predict other areas at risk for malaria reemergence in Greece. Our data sources were countrywide georeferenced environmental and climatic information, all acquired from the European Environment and Epidemiology Network data repository and prepared for spatial modeling (*7*). Variables considered were temperature, vegetation seasonal variations, altitude, land-cover categories, and demographic indicators. Daytime and nighttime Land Surface Temperature and Normalized Difference Vegetation Index were retrieved from the 1 km–resolution long-term Temporal Fourier transformed imagery from Moderate Resolution Imaging Spectroradiometer (*8*). Altitude values were derived from the Global Land One-km Base Elevation Digital Elevation Model data from the US Geological Survey Earth Resources Observation Systems data center (http://eros.usgs.gov). The coordination of information on the environment (CORINE) land cover from the European Environment Agency provided the framework for land cover patterns exploratory analysis (www.eea.europa.eu/data-and-maps/data/corine-land-cover-2000-clc2000-seamless-vector-database-3). The population density grid of the Joint Research Centre was used as demographic indicator (www.eea.europa.eu/data-and-maps/data/population-density-disaggregated-with-corine-land-cover-2000-1).

The spatial scale for this study was a polygon equivalent to a circular buffer of 3.5 km (40,000 ha). This spatial resolution was considered appropriate to the relatively fine scale at which environmental variability can affect malaria transmission. The construction of the land-cover variables is described in the Technical Appendix. A disease risk map was generated by using nonlinear discriminant analysis available in eRiskMapper version 1.1.4 (www.tala.ox.ac.uk) and is described in detail in the online Technical Appendix.

**Figure 1:**
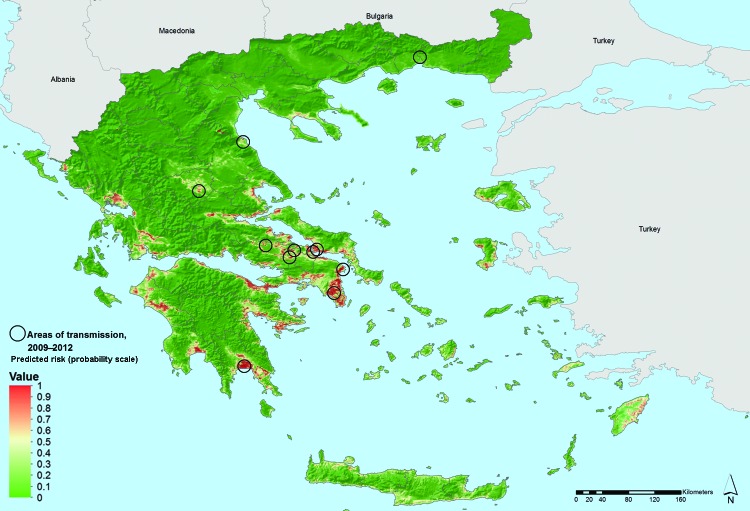
Areas latently hospitable and environmentally permissive for persistent malaria transmission, Greece, 2009–2012. Map showing areas predicted to be environmentally suitable for malaria transmission. Values from 0 to 0.5 (dark to light green) indicate conditions not favorable for malaria transmission (based on locally acquired cases); yellow to dark red areas delineate conditions increasingly favorable for transmission (values from 0.5 to 1).

The first 10 best ranked variables comprised sea-level altitude and land-surface temperature (Table). Parameters of nighttime and daytime temperatures were predictive in this model; the annual variation (amplitude) and the mean absolute temperature values scored high in the ranking. Predicted suitability of areas for persistent malaria transmission based on these variables are characterized by low elevation; warmer temperatures (which might enable more rapid mosquito and parasite development); and intensive, year-round irrigated agriculture with complex cultivation patterns (generally requiring a high degree of manual labor) (Figure 1, Appendix). These are probable contributing factors to mosquitoe presence and, possibly, to malaria transmission.

**Table:** Environmental suitability mapping of malaria, Greece, 2009–2012*

Variable†	Rank
Digital elevation model	4.4
LST day amplitude 2	5.5
NDVI phase 2	7.0
LST nighttime amplitude 2	7.3
LST nighttime mean	8.1
LST day, maximum	8.1
LST day, mean	8.3
LST day phase 2	8.6
NDVI, maximum	8.7
LST night, maximum	8.9

*Classification of 10 variables average rank (1 to 10) for all bootstrapped cycles. Data sources are as follows: Global 30 Arc-Second Elevation Dataset (GTOPO30), 2005 (http://eros.usgs.gov); CORINE Land Cover 2000 seamless vector data, version 15 (Aug 2011), European Environment Agency, 2011 (www.eea.europa.eu/data-and-maps/data/corine-land-cover-2000-clc2000-seamless-vector-database-3); Raster data on population density using CORINE Land Cover 2000 inventory, European Environment Agency. 2009 (www.eea.europa.eu/data-and-maps/data/population-density-disaggregated-with-corine-land-cover-2000-2).  †LST, Land Surface Temperature; NDVI, Normalized Difference Vegetation Index.

**Figure 2:**
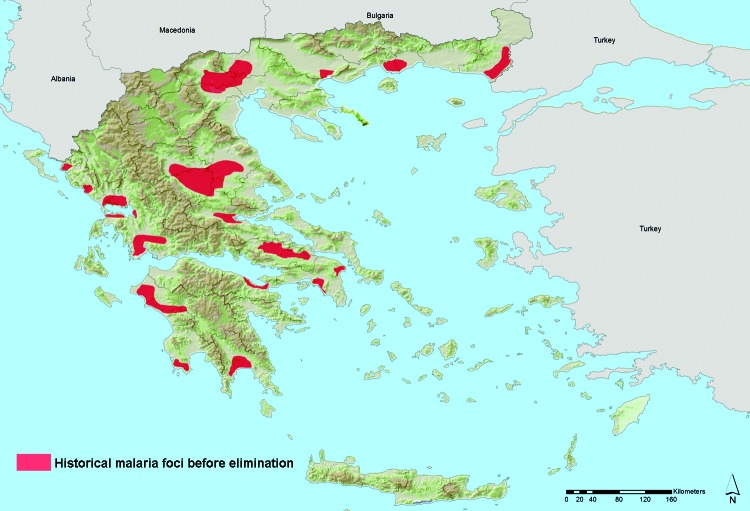
Areas of historic malaria transmission before elimination, Greece. Greece was officially declared malaria free in 1974, after a national malaria elimination effort during 1946–1960. Data sources: adapted from (*10*; Ministry of Health. Map of confirmed laboratory species–1952, unpub. data).

The accuracy statistics of the bootstrap model revealed a sensitivity of 0.98 (where 1 denotes recognition of all actual presences) and specificity of 0.98 (where 1 denotes recognition of all actual absences). Moreover, historical presence of malaria in Greece, before disease elimination, was extracted from several sources and showed a partially coincident distribution pattern with the suitability map: 41% overlap in Peloponnese, 63% along the west coast of central Greece and Epirus, and 39% in eastern central Greece (Figure 2, Appendix). The best overlap was found along the west coast, but in the northern part, the model did not match the historical pattern very well. This discrepancy might be explained by the low rank of landscape pattern variables obtained into nonlinear discriminant analysis modeling.

## Conclusions

We assessed environmental and climatic characteristics of the areas with autochthonous spread of *P. vivax* malaria in Greece during 2009–2012 and delineated similar areas possibly suitable for transmission in Greece. Sea-level altitude and the mean and annual variation of land-surface temperature for daytime and nighttime were predictors in our model.

A major limitation of our study is that it considers only environmental suitability for transmission, not risk for transmission per se. Our approach did not account for certain factors in this regard, for example, presence/absence of malaria vectors under collection in 2012. This partly reflects the lack of knowledge about what mosquito species were responsible for recent outbreaks, although historic work suggests *Anopheles sacharovi*, *An. maculipennis s.s.*, and *An. superpictus* (*9*,*10*). Indeed, 2 of these species, *An. sacharovi* and *An. superpictus*, have been implicated as the probable dominant vectors throughout Greece and adjacent areas (*11*). Hence, species would seem to be reasonable targets for any mosquito surveillance efforts.

Our model did not highlight areas of northern Greece previously associated with malaria (*4*,*5*) as being particularly suitable for transmission. Further refinement of our approach, perhaps including vector information, might address this shortcoming. Further study on the malariogenic potential should be conducted to complete our assessment. Despite these limitations, this spatial analysis can be useful and can help guide the public health response to threats, such as malaria, by directing surveillance and control activities and/or by identifying uncertainties relevant to disease risk mapping and response.

Model accuracy and public health practice can be improved through vigilant disease and vector surveillance with timely case notification (*12*). Yet, despite these potential limitations, we believe that this spatial analysis is a useful tool; it can help guide response(s), integrated preparedness and response activities, including targeted epidemiologic and entomologic surveillance, vector control activities, and awareness rising among the general population and health care workers, in the areas environmentally suitable for transmission.

Technical Appendix Description of the construction on land-cover variables, comparison of nonlinear discriminant analysis and historical maps, and nonlinear discriminant analysis.
