# Supplementary material for: Mapping Environmental Suitability for Malaria Transmission, Greece
Source: Emerg Infect Dis. 2013 May;19(5):784–6. doi: 10.3201/eid1905.120811 (PMC3647495; doi:10.3201/eid1905.120811)
Supplement: Technical Appendix — Description of the construction on land-cover variables, comparison of nonlinear discriminant analysis and historical maps, and nonlinear discriminant analysis. [file 12-0811-Techapp-s1.pdf]

Article DOI: http://dx.doi.org/10.3201/eid1905.120811

Mapping Environmental Suitability of Malaria Transmission, Greece

Technical Appendix

Description of the Construction on Land-cover Variables

For land cover, variables were constructed by aggregating level 3 CORINE Land Cover classifications (www.eea.europa.eu/data-and-maps/data/corine-land-cover-2000-clc2000-seamless-vector-database-3). Associations of these classifications with malaria data were then assessed through logistic regression by using STATA 10.1-SE (StataCorp LP, College Station, TX, USA) by fitting against the presence/absence of cases. Next, land-cover classifications that were significantly predictive ($p<10^{-3}$) were pooled to form additional informative layers, representing agricultural and water source patterns. The agricultural layer resulted from an aggregation of different classes, namely fruit trees; berry plantations, and olive groves; land principally occupied by agriculture with significant areas of natural vegetation; and annual crops associated with permanent crops and complex cultivation patterns. Water sources were represented by stagnant water or wetlands, permanently irrigated lands, water bodies, coastal lagoon, estuaries, and salt and inland marshes. Artificial surfaces, forest and seminatural areas classes were not significant and were excluded from further analysis.

Nonlinear Discriminant Analysis

Nonlinear discriminant analysis requires both presence and absence data; however, surveillance data seldom record absences and, if so, seldom in sufficient numbers. “Pseudo-absence” data points must first be generated from the presence data; the models then aim to discriminate between these 2 categories by using the predictor variables available. For the mathematical modeling, besides the 69 domestic malaria case presence points, 210 pseudo-absence points were randomly generated with a minimum distance of 3.5 km from presence points. A disease risk map was generated with these presence and pseudo-absence points, by using nonlinear discriminant analysis (NLDA) available in eRiskMapper version 1.1.4 (www.tala.ox.ac.uk).

Euclidean distance layers from the newly generated landscape classes (distance from agriculture, water sources, and sea coastline) were computed by using ARCGIS Desktop 10 (ESRI, Redlands, CA, USA) and subsequently inputted as additional predictors in NLDA. The NLDA was conducted with 100 bootstrap cycles with 20 replacements for pseudo-absence points in each cycle. To ensure the assumption of multivariate normality of each input variable distribution, clustering was done for absence points by using the best ranked parameters.

Due to the high number of islands in Greece that strongly affect the distribution of the variable “distance-from-coastline,” a sensitivity analysis was performed with and without an islands mask. The pairwise correlation coefficient of a random sample of 20 000 points with a continental location between the 2 models was 0.84 ($p<10^{-4}$).

Comparison of NLDA and Historical Maps

A comparison between the NLDA and the historical risk maps is hampered by the fact that both maps are probably incomplete and should not be regarded as a gold standard. For a high-resolution visualization, please see the 4 panels in the Technical Appendix Figure.

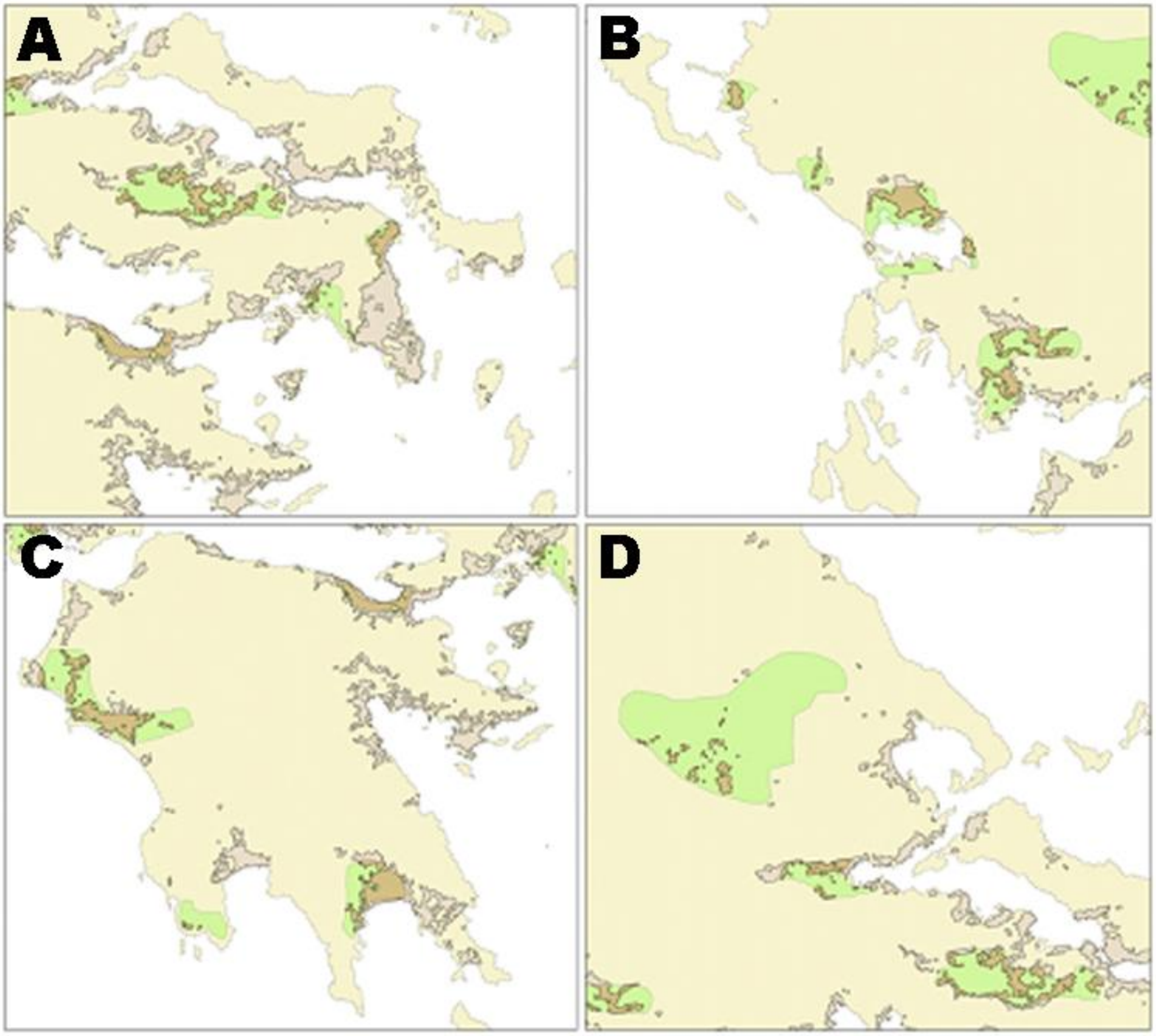

Technical Appendix Figure. Comparison of the historical map with Athens and East/Central Greece (A); Epirus, East/Central Greece (B); Peloponnese (C); and South Thessaly (D). Light brown, Greece; green, historical area; light violet, NLDA model; deep brown, overlapping areas between historical and nonlinear discriminant analysis model.
